# Laughter and humor as complementary and alternative medicines for dementia patients

**DOI:** 10.1186/1472-6882-10-28

**Published:** 2010-06-18

**Authors:** Masatoshi Takeda, Ryota Hashimoto, Takashi Kudo, Masayasu Okochi, Shinji Tagami, Takashi Morihara, Golam Sadick, Toshihisa Tanaka

**Affiliations:** 1 Department of Psychiatry, Osaka University Graduate School of Medicine, Suita, Japan

## Abstract

**Background:**

The number of dementia patients has increased worldwide, with an estimated 13.7 million dementia patients in the Asia Pacific region alone. This number is expected to increase to 64.6 million by the year 2050.

**Discussion:**

As a result of advances in research, there several pharmacological therapies available for the treatment of dementia patients. However, current treatments do not suppress the disease process and cannot prevent dementia, and it will be some time before these goals are realized. In the meantime, complementary and alternative medicine (CAM) is an important aspect in the treatment of dementia patients to improve their quality of life throughout the long course of the disease. Considering the individuality of dementia patients, applicability of laughter and humor therapy is discussed. Even though there are many things that need to be elucidated regarding the mechanisms underlying the beneficial effects of laughter and humor, both may be good CAM for dementia patients if they are applied carefully and properly.

**Summary:**

In this debate article, the physiological basis and actual application of laughter and humor in the treatment of dementia patients are presented for discussion on the applicability to dementia patients.

## Background

Because of the rapidly increasing elderly population, the need for psychogeriatric services will increase in coming years. In particular, a faster aging of the population has been observed in Asian countries compared with that in Western countries. The World Health Organization has proposed that for a society to be called 'aging', the proportion of elderly citizens (aged 65 years and older) must be 7%. Once this proportion reaches 14%, a society becomes an 'aged society' [1]. It took 24 years for Japan to move from an aging society (in 1970) to an aged society (in 1994); in comparison, in most Western countries this process takes 60-120 years [1]. Korea is expected to become an aged society by 2019, only 19 years after becoming an aging society (2000).

Considerable progress has been made in psychogeriatric services as a result of increased knowledge of brain science, neuroscience, molecular genetics, brain imaging, and many other new technologies [2]. The mechanisms underlying the cognitive impairment in dementia patients are now understood because of findings from brain science and neuropsychological investigations [3,4]. Electrophysiology (e.g. electroencephalography topography, event-related potentials (ERP), and magnetoencephalography (MEG), brain imaging (e.g. magnetic resonance imaging (MRI), single photon emission computed tomography (SPECT), positron emission tomography (PET) and even newer technologies, such as near-infrared spectroscopy (NIRS) and magnetic resonance spectroscopy (MRS), are versatile tools available to confirm psychogeriatric diagnoses [5]. Furthermore, genetic information is routinely used to evaluate the risk, as well as the prognosis, of a disease and a patient's response to drug treatment [6].

Treatment of behavioral and psychological symptoms of dementia (BPSD) remains one of the most unmet needs in psychogeriatrics [7,8], with more effective pharmacological [9,10] and non-pharmacological interventions [11-13] needed. Psychogeriatrics is, however, a clinical subspecialty in which treatment should be directed towards the person as a whole. Consideration of the person and holistic care are essential, including a bio-psycho-socio-ethical evaluation of each patient, because the life of the elderly is so different [1]. Furthermore, psychogeriatric services can be applied to patients in the pre-stages of dementia, including those with mild cognitive impairment (MCI) [14,15] and subjective cognitive impairment (SCI) [16]. Dementia patients, including those with MCI and SCI, can benefit from psychogeriatric services, and the specific application of laughter and humor therapy in the treatment of these patients is discussed in the present article.

### Dementia patients require individualized and life-long intervention

In 2005, it was reported that there were 13.7 million dementia patients in the Asia Pacific region alone and that this number is expected to increase to 64.6 million by the year 2050, a 4.7-fold increase in just 45 years [1]. In addition to its high prevalence, the considerable disruption to patients' daily lives, the burden to caregivers, and the long duration of the disease make dementia, especially Alzheimer's disease (AD), the most malignant disease of our time.

The symptoms of AD differ between individual patients. At the onset of dementia in some patients, certain personality traits that had been well controlled in the past become accentuated, whereas in others there is a 'loss of personality', where the uniqueness of the patient's personality is lost. Some patients show a more rapid deterioration of cognitive function, whereas others show a slower rate of cognitive decline. Some patients exhibit various types of BPSD, whereas others exhibit few abnormal behaviors [7]. Furthermore, the physical, personal, familial, economic, and social environments differ between patients. Thus, each patient should be evaluated as an individual in terms of his/her needs for intervention, taking into account previous social functioning, family structure, and the patient's living environment in order to deliver the most appropriate care. Interventions for dementia patients need to be individualized further taking into consideration the different genetic and environmental factors that are specific to each patient.

The premorbid mental capacity differs between subjects and the symptoms exhibited by dementia patients vary quite widely. Considering the difference in symptoms of dementia patients, a more individualized treatment and management program should be considered taking into account of the emotional and affective responses of each patient individually. In this respect, the possibility of using laughter and humor therapy as a complementary and alternative medicine (CAM) for the treatment of dementia patients is discussed below.

## Discussion

### Laughter as a CAM

Although modern medical science has enabled correct diagnoses to be made and proper treatments to be initiated for acute diseases caused by exogenous pathogenic factors, there are still numerous chronic, incurable diseases caused by endogenous factors, such as cancer, dementia, hypertension, diabetes, chronic pain etc., for which there is no effective treatment, leaving patients with these conditions to suffer. To facilitate the better management of these chronic diseases, recent attention has focused on the use of CAM, together with Oriental and traditional medicines [17]. CAM is defined by the American Cancer Society as '...supportive methods used to complement evidence-based treatment. Complementary therapies do not replace mainstream treatment and are not promoted to cure disease. Rather, they control symptoms and improve well-being and quality of life'[18]. In contrast, alternative therapies, or alternative medicine, involve non-mainstream treatments that are sometimes used by patients instead of orthodox treatments. Examples of CAM include music therapy, drama therapy, aromatherapy, animal-assisted therapy, gardening, horse riding, exercise, bathing, herbal medications, acupuncture, moxibustion, shiatsu, and yoga among others [19]. However, these therapies have not been well defined. Some are simply based on legend or belief, whereas others are traditionally applied but without any scientific basis.

It is widely accepted that a patient's emotional state will affect the course of the disease. Human emotional behavior can be either negative or positive. Negative emotional behavior is accompanied by disgust, fear, or alarm, which induces a prompt, narrowed response to the stimulus responsible for the life crisis. The 'fight-or-flight' response is the general outcome of negative emotions, in which the sympathetic autonomic nervous system is dominant. Conversely, in safe and relaxing situations, positive emotional behavior is associated with joy, play, and humor, with predominant functioning of the parasympathetic system, which induces responses of extended open behaviors that are helpful in learning new behavioral patterns. Laughter associated with a pleasant feeling is often observed under positive emotional conditions.

Laughter has a unique position in CAM. The benefits of laughter have been recognized historically. As stated by Bertrand Russell, 'Laughter is the most inexpensive and most effective wonder drug. Laughter is a universal medicine'. Laughter has been regarded as beneficial for human health for a long time, with some of the benefits attributed to laughter including improved immunological [20] and endocrinological [21] responses and increased pain tolerance [22]. Laughter therapy, humor therapy, laughter meditation, and laughter clubs all have unique implications as group programs and as self-management techniques. For practitioners to implement credible programs and effectively teach self-management techniques, further empirical research on the physical, psychosocial, and placebo effects of laughter and humor needs to be conducted.

### Physiology of laughter and smiling

Speech and laughter are unique to humans. Although there is considerable information regarding the neuronal representation of speech, little is known about the neural mechanisms of laughter. As described by Charles Darwin, laughter, which is a ubiquitous and unique maneuver of humans that results in a totally defenseless posture involving movement of such a wide area of musculature, should have some beneficial meaning in terms of the evolution of this species [23]. Laughter should mean a lot to our lives.

Newborn babies smile within the first 5 weeks after birth and laugh within the first 4 months. Some smiles are voluntary and smiling can be differentiated into 16 different expressions [24], but there is only one expression of laughter. When we smile, the mouth angles are lifted and the orbits of the eye become thin and surrounded by wrinkles as a result of the simultaneous contraction of the muscularis zygomaticus major and orbicularis oculi. In addition to these muscles, when a person is laughing a wider area of the musculature, including facial, pharyngeal, and respiratory muscles, is simultaneously contracted [24].

Laughter and smiling are usually produced as a message of good will to others. In primates, facial expressions showing bared teeth mean friendliness and primates use these expressions to transmit their sociability and the fact that they have no hostile feelings. Because some forms of smiling are voluntary and easily faked, laughter, which requires a more synergetic contraction of the wider musculature, is believed to have evolved in humans to express a secure, safe message to others.

### Neural circuits of laughter and smiling

Laughter is the physiological opposite of crying and is usually an expression of happiness involving typical facial movements and contractions of the respiratory muscles [25]. Neural correlates for laughter may include the anterior cingulate gyrus, which provides emotional consciousness to an individual's experience and is partially under the control of the frontal cortex [26]. The caudal hypothalamus is also involved, acting as the center coordinating emotional changes, including laughter, whereas the temporal amygdala may provide emotional coloring to perceptions and aid in understanding humor [26,27]. Finally, the ventral pontomedullary center for laughter coordinates facial expressions, expirations, and emotional vocalization.

The expression of laughter depends on two partially independent neuronal pathways. One is the 'involuntary' system involving the amygdala, thalamic, hypothalamic and subthalamic areas, and the dorsal brain stem [27]; the other is 'voluntary' and originates in the premotor opercular areas, leading through the motor cortex and the pyramidal tract to the ventral brain stem.

The neural circuit underlying laughter may have three main brain components: (i) cognitive areas, such as sections of the frontal lobe that help a person understand the situation; (ii) a movement area (probably the supplemental motor area) that triggers muscle movements to induce a smile or laughter; and (iii) an emotional component that actuates the perception of happiness after an amusing experience, possibly facilitated by the nucleus accumbens [28].

### Neural circuits of humor

Humor can be broadly defined as 'something that is, or is designed to be, comical or amusing'. More specific definitions vary, but humorous communication certainly causes increased feelings of happiness and laughter in those who respond to it, whether due to witty comments or amusing behavior.

Freud's psychodynamic viewpoint described humor as the strongest form of the defense mechanism that allows an individual to face problems and avoid negative emotion [29]. Humor is believed to be effective in distancing oneself, framing problems with perspective, and proactively managing distress [30-32].

Although physiological research on the effects of humor on the body is only just developing, there may be quantifiable health care benefits of humor. Research involving additional measurements of a sense of humor, including self-reported instruments, peer ratings, and comedy monologues, suggests that humor moderates the impact of stressful life events on mood disturbances, such as depression and anxiety, salivary immunoglobulin, and positive affect [33-35]. Similar moderating effects of humor have been identified for depression, insomnia, loneliness, and self-esteem, although not for anxiety [36-39].

Good humor makes people laugh just like pain makes people cry, but humor requires complex neural circuits. Humor is perceived at the beginning as surprise or disharmony, then the paradox is solved, and, finally, the punch line is understood in association with a pleasant feeling. The appreciation of humor requires a wide area of neural circuits covering attention, working memory, flexible thinking, extraction of word meaning, and positive mood. Patients with lesions in the right frontal lobe have difficulty appreciating humor because of impaired integration of cognition and emotion. Different brain areas are activated by jokes/puns and comics [40]. Humor is present in any social situation, and the nature of what is perceived as amusing varies widely among individuals, societies, and cultures. Everyone enjoys laughing, but a misjudged humorous comment can cause offense, so although laughter is almost always positive, humor itself can provoke mixed emotional responses.

### Classification of laughter and smiling

Laughter and smiling can be classified into one of three categories based on evolutional staging as follows: (A) that evoked by a release of tension; (B) that associated with pleasant feelings; and (C) that used for social communication (Table 1).

**Table 1:** Relationship between laughter/smiling and the progression of dementia

Type of laughter/smile	Preservation in dementia	
	Early stages	Advanced stages
A1. Release from strong tension	+	+
A2. Release from weak tension	+	+
B1. Fulfillment of instinctive needs	+	+
B2. Fulfillment of expectations	+	-
B3. Feelings of superiority	+	-
B4. Feelings of disharmony	+/-	-
C1. Cooperative	-	-
C2. Defensive	-	-
C3. Aggressive	-	-
C4. Devaluating	-	-

The type of laughter and/or smiling can be classified into one of three categories: (A1,2) that evoked by a release of tension; (B1-4) that associated with pleasant feelings; and (C1-4) that used for social communication. Laughter and smiling induced by a release of tension is regarded as the most basic type and is preserved as the phylogenetically primitive type. Laughter and smiling associated with pleasant feelings has developed with the evolution of humans. Laughter and smiling as communication tools are the most sophisticated and have developed with the sociability of humans. Dementia patients lose the ability to laugh and smile as the disease progresses. Laughter and smiling as communication tools may be lost in the early stages of dementia, when the clinical symptoms of dementia appear. Of the different forms of laughter and smiling associated with pleasant feelings, those induced by disharmony may be lost in early stages of dementia because of the cognitive impairment that may limit a patient's understanding. However, laughter and smiling induced by feelings of superiority, fulfillment of expectations, and fulfillment of instinctive needs are preserved until the advanced stages of dementia. Laughter and smiling in response to a release of tension are preserved in most dementia patients.

Laughter or smiling caused by a release of tension is the most basic biological form, and occurs spontaneously in an individual who experiences release from a strenuous tension. The purpose of laughter in this context has been hypothesized to be the release of inner energy accumulated in response to the stress [23]. Laughter to relax is important for the maintenance of mental health. Long-lasting mental tension is accompanied by a hyperaroused state of the sympathetic nervous system, which can be released by laughing [24]. From the viewpoint of mental health, laughter evoked in response to the release of tension is the most important.

The second category, laughter that is provoked or accompanied by pleasant feelings, can be further subdivided into laughter caused by: (B1) fulfillment of instinctive needs; (B2) fulfillment of expectations; (B3) a feeling of superiority; and (B4) recognition of mix-ups. As early as 5 weeks after birth, babies smile after feeding. This is the first laughter observed in human life, elicited by a fulfillment of instinctive needs. Similar laughter is observed in adults after a good meal or a good sleep. When our expectations are realized, especially after hard work and/or endeavor, we usually laugh in association with pleasant feelings, which can be amplified by colleagues sharing in our achievement, with the most explosive form of laughter then being observed. Laughter caused by a feeling of superiority is a type of scornful laughter or a cold smile that has been proposed by some researchers to be the prototype of laughter [23]. Laughter associated with disharmony and/or mismatch is caused by simple mistakes or funny happenings that cause no harm. This sort of laughter can be elicited only when the disharmony is sudden, unexpected, and the results of the misunderstanding are harmless.

The third category of laughter is that used as a communication tool. Facial expressions are important components of laughter and we use these expressions to transmit our intention to be friends with others. Laughing and smiling used to communicate with others can be further subdivided into laughter and smiling for cooperation, defense, aggression, and devaluation. A typical example of cooperative smiling is that used as a greeting. We usually say hello and shake hands while smiling. A defensive smile can be observed when someone is trying to conceal their inner feelings, whereas aggressive laughter can also be called scornful laughter. Everyone dislikes being laughed at and, consequently, aggressive laughter is extremely powerful. Smiling to devalue something is often used in daily life; for example, when the train door shuts in our face, we often give a wry smile to cancel out the impact of the event.

### Laughter in dementia patients

Laughter is usually provoked or accompanied by positive emotions. In clinical settings, it is always desirable for patients, their families, and staff to share relaxed and happy feelings, because patients are often under continuous strain and enormous pressure as a result of their illness. The more serious the illness, the more overwhelming the strain to the patients and their families. Dementia patients are usually under considerable strain, at least at the beginning of their illness. Patients' families are placed under even more stress because of the burden of care [41]. A positive emotion, together with laughter, may enable dementia patients to cope with their illness better, improve immune function, increase pain tolerance, and decrease the stress response. When a positive attitude is shared by patients and staff, it can have a positive effect on the emotional-affective and cognitive functioning of the patients [42,43].

Because the social life of dementia patients is impaired by their illness, they can easily feel isolated. Thus, a feeling that unites them, or provides some sort of bond, with their family and the community can be very beneficial. Dementia patients are often encouraged to participate in daily activities with other people and the positive emotions that are shared by the patients and the care staff help the patients maintain social contact.

Several psychosocial interventions are applied to dementia patients in clinical settings [44]. Examples include cognitive rehabilitation, reminiscence therapy, art therapy, drama therapy, and aerobic exercise [45]. In these activities, a positive attitude of patients is essential and it is always true that a greater effect can be expected when patients participate willingly with a positive outlook. In the case of cognitive rehabilitation, active participation is the condition under which good outcomes can be expected. If the patients are reluctant to participate in the activities, it is unlikely that the program will have any beneficial effects.

Dementia patients become anxious and irritated because they are unable to glean sufficient information from their surroundings due to their impaired cognitive functioning [46]. They are easily trapped in a state in which they feel unsafe, alarmed, and insecure, which, in turn, reduces their ability to process information from their surroundings. With even less secure information, they become more alarmed, leading to negative emotional behavior.

Dementia patients often show various types of BPSD during the course of their illness. Aggression, refusal to cooperate, negativity, and apathy are common, all of which contribute to the further isolation of these patients. In this sense, it is important to keep patients with BPSD within the community.

Because BPSD can often be the most formidable barrier to the care of dementia patients, it is highly recommended that the occurrence of BPSD is prevented. To reduce the occurrence of BPSD in dementia patients, patients should be kept in a stable and safe environment, efforts should be made to ensure good communication with the patients, and patients should be kept feeling relaxed and safe. By doing so, the patients are more likely to laugh and smile.

It is true that laughter and smiling decrease over time in most dementia patients, but it is important to note that not all forms of laughter and smiling are equally reduced. The ability to laugh for social communication is readily lost by dementia patients at the onset of their illness, concomitant with the loss of a social life and their ability to process information, but laughter in response to the release of tension is preserved until the advanced stages of the disease. When dementia patients are released from either physical or mental strain, they always smile. Laughter caused by feelings of disharmony is not usually preserved in dementia patients because of impaired cognitive functioning and because these patients are no longer able to understand the meaning of complicated situations, which means they often cannot understand the punch lines of jokes or appreciate humor.

As discussed above, laughter associated with pleasant feelings can be further subdivided into four types, fulfillment of instinctive needs, fulfillment of expectations, a feeling of superiority, and recognition of mix-ups. Most laughter associated with pleasant feelings is preserved in dementia patients, with observations indicating that these patients laugh and smile when they are exposed to pleasant stimuli. They smile when they are well fed and when they have had a good sleep. They also smile and laugh when they have attained self-set goals. Laughter associated with feelings of superiority is clearly preserved in most dementia patients; they become happy and pleasant when their superiority is recognized. Conversely, when these patients feel humiliated, they become angry and insulted.

Thus, the basic form of laughter is preserved in dementia patients, but the social form of laughter is sometimes lost in the advanced stages of the disease. It is important to ensure that dementia patients are kept in a safe and relaxed environment (and not in alarmed and tensioned), which will make it more likely that these patients will be able to laugh and smile.

### Humor in dementia patients

Humor has positive physiological and psychological effects in a variety of situations. The psychiatric literature purports humor as an effective tool in psychiatric illness and psychotherapy. Benefits of humor in business, management, education, and clinical settings are widely recognized because the right perspective facilitates problem solving both interpersonally and in a group setting. Furthermore, humor puts people at ease, promoting the expression and exchange of ideas. Not only can humor benefit patients, but the use of humor can facilitate the effective management of staff and others in the health care setting [22].

Humor is delicate and sensitive by nature. Humor can be properly appreciated when it is expressed in the right time, right place, and on the right occasion. Confidence, or trust, between the sender and receiver is an important aspect of humor. Establishing this trust is a prerequisite for the introduction of appropriately timed humor. No humor can be appreciated by patients when there is no trust between the patient and care staff. If one side is defensive or angry, he/she may find that the use of humor by the other party is offensive or insulting [47,48]. Patients may also become upset about jokes made at their expense, fearing humiliation and stigmatization [49]. The appropriateness of humor depends on the culture, education, and cognitive function of the receiver. Therefore, the use of humor must be timed wisely and it must be used carefully.

Dementia patients may be more sensitive to jokes or humor than healthy people because patients in the early stages of the disease know that they have difficulties understanding complicated things. Dementia patients with cognitive impairment have difficulty appreciating the disharmony in information sent as humor. Humor should be presented to dementia patients after close evaluation. There are no definitive rules, but humor should generally be introduced slowly; if there is no response or the response is negative, it may be a good idea to abandon all attempts to introduce humor, at least during that clinical encounter [50]. Humor can be used as a defense mechanism in an adverse setting and has obvious value for dementia patients if it is properly addressed and accepted. But the impaired cognitive function of dementia patients must be kept in mind so that humor is presented at the right time, in the right place, and on the right occasion. Everyone enjoys laughing, but a misjudged humorous comment can cause offense, so although laughter is almost always positive, humor itself can provoke mixed emotional responses.

The other reactions--anger, depression, suppression, denial--took a little piece of me with them. Each made me feel just a little less human. Laughter made me more open to ideas, more inviting to others, and even a little stronger inside. It proved to me that, even as my body was devastated and my spirit challenged, I was still a vital human being. Scott Burton [51]

## Summary

Dementia patients should be cared for taking into consideration their individual capacities, which differ from patient to patient. Most laughter and smiling is preserved in dementia patients until the end of the clinical course, even though laughter and smiling as a means of communication is lost during the early stages of the disease. Laughter and smiling associated with pleasant feelings, with the exception of laughing in response to feelings of disharmony, and laughter induced by the release of tension can be used in the treatment of dementia patients. The use of humor, covering issues of the fulfillment of instinctive needs and expectations, as well as feelings of superiority (Table 1), can be a good and effective complementary and alternative intervention in the treatment of dementia patients.

## Competing interests

The authors declare that they have no competing interests.

## Authors' contributions

MT, TK, and TT discussed the importance of laughter and humor to dementia patients and drafted the manuscript. MO, ST, and TM searched for the data on the topics in the literatures. MT, RH, and GS devised the table. All authors have read and approved the final manuscript.

## Pre-publication history

The pre-publication history for this paper can be accessed here:

http://www.biomedcentral.com/1472-6882/10/28/prepub
